# A Case of *Plasmodium Falciparum* Malaria Presentation

**DOI:** 10.1097/MD.0000000000001415

**Published:** 2015-08-28

**Authors:** Osman Nawazish Salaria, Salman Nawazish Salaria, Riyad Basir, Misbahuddin Khaja

**Affiliations:** From the Lincoln Medical and Mental Health Center, New York, New York, USA.

## Abstract

New York City is a multicultural city where people of different ethnicities and backgrounds from all over the world live together. Of the different ethnicities, it is home to a large population of Western African immigrants. This case report is that of an elderly female of Western African descent presenting to Lincoln Hospitals Emergency Department with fevers and fatigue.

The patients travel history to Togo, along with her symptoms, resulted in a differential diagnosis which included Ebola as well as Malaria. New York City's Department of Health and Mental Hygiene was contacted for further clarification of presence of Ebola in Togo. The present case report is meant to educate about the presentation, hospital course, and differential diagnoses of a patient traveling from Western Africa with fever and chills.

## INTRODUCTION

Malaria is a frequent parasitic infection prevalent in Africa. Around 300 million are infected annually in Africa by malaria and 1 to 2 million will die from the disease.^1^ Of the 4 human parasitic species that have been identified, *Plasmodium falciparum* has been known to cause significant morbidity and mortality, particularly in children and pregnant women.^1^ Strategies to counteract malaria incidence, such as community health workers outreach and insecticide treated nets have been instituted in recent years; however, their effect has not been of much significance.^2^

Ebola virus disease has caused much concern with its global rise in incidence and prevalence recently. The current epidemic which has centered mainly in Western African nations of Guinea, Sierra Leone, and Liberia has now spread outside of borders of Africa to involve the United States.^3^ Much of the presenting symptoms and signs of the disease mimic other diseases such as typhoid fever and malaria.^3,4^

There is much overlap between presentations of both *P. falciparum* malaria and Ebola virus disease. Without confirmatory blood tests searching for malaria parasites or viral RNA and viral antibodies a diagnosis is very difficult to achieve.

## CASE REPORT

A 67 y/o (year old) female from Western Africa initially presented to the Emergency Department (ED) complaining of fatigue and subjective fevers for the past 2 days. Patient complained that her fevers were associated with headaches, but not chills, rigors, or chest pain. Index of suspicion for malaria was high as patient had recently traveled from an endemic region. Patients travel history to Western Africa and the presenting symptoms also made us consider a possibility of Ebola virus disease.

Past medical history included diabetes, hypertension, and a history of recent travel to her home country of Togo for 5 months. Patient had returned 5 days ago from her travel and started to develop symptoms of fevers and fatigue. Patient denied any immunizations received before traveling. Past surgical history included a left breast mastectomy done back in France 1987. Medication history included Amlodipine, Aspirin, Calcium Carbonate, Synthroid, Pioglitazone, Humalog, Glucovance, Crestor, Januvia, and Lisinopril. After initial presentation to the ED for 2 days of fevers and fatigue, she was accepted by Medicine and transferred to the general medical floors. The patient had a blood pressure of 123/55, pulse of 86 beats per minute (bpm), Temperature of 98.5 °F, and respiratory rate of 16 at the time of admission. Physical examination did not disclose any specific abnormalities.

**FIGURE 1:**
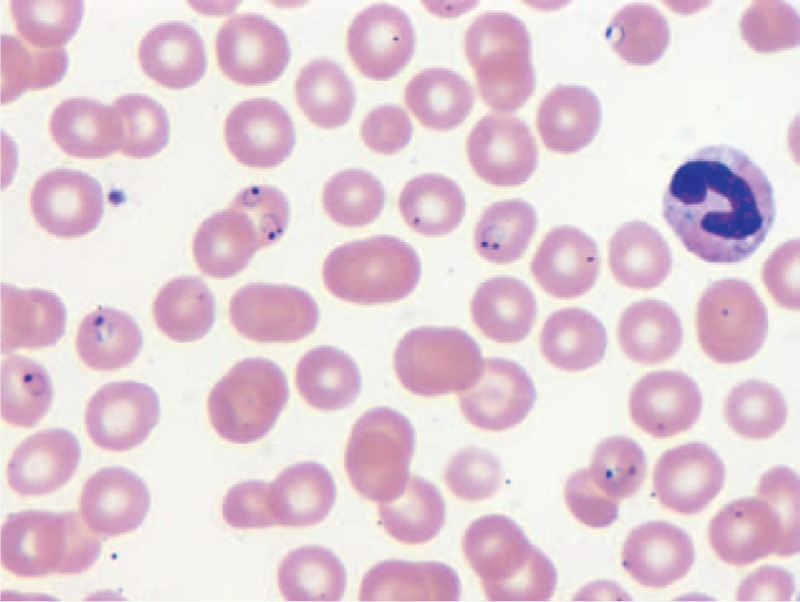
Peripheral blood smear of patient on initial presentation.

**FIGURE 2:**
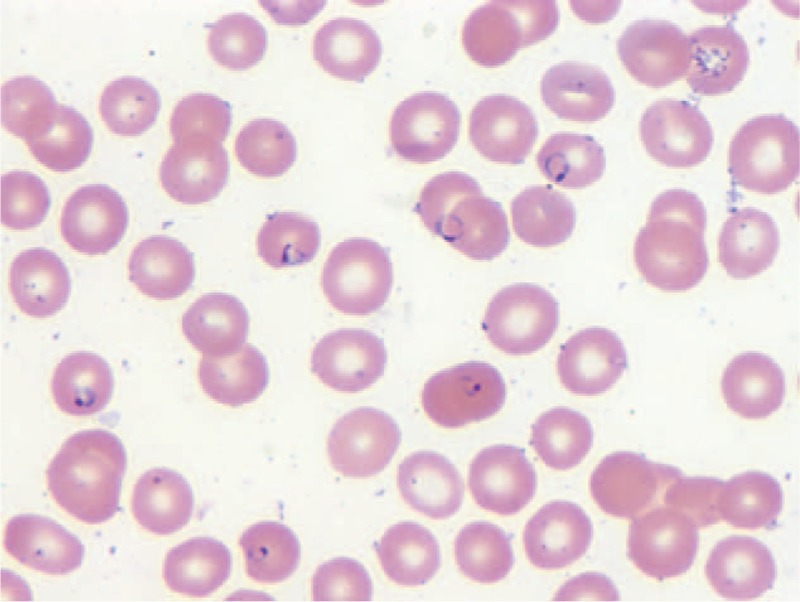
Peripheral blood smear of patient on day 3.

**FIGURE 3:**
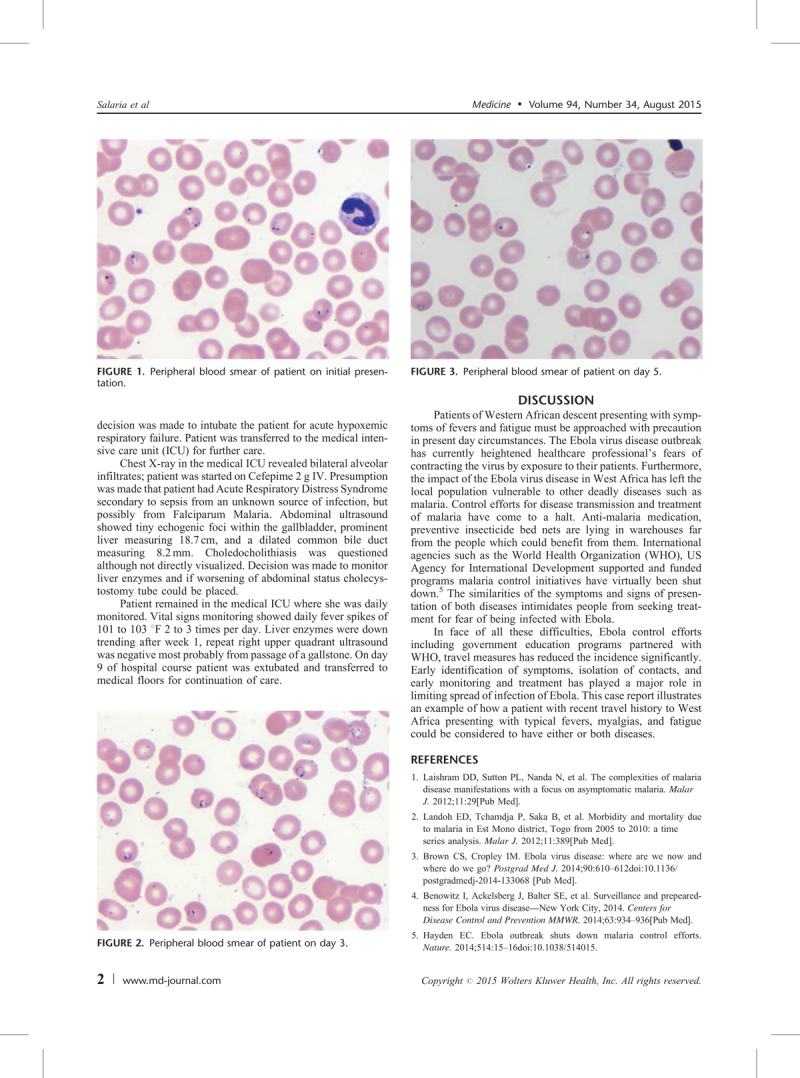
Peripheral blood smear of patient on day 5.

Labs including complete blood count, chemistry, liver function tests, malaria peripheral smears, and reitculocyte level were withdrawn from the patient. Patient had white blood cell (WBC) count of 12.6, Hb (hemoglobin) 10.7, Hct (hematocrit) 30.6, Plt (platelet) 80, BUN (blood urea nitrogen) 12, Creat (creatinine) 0.3, and blood glucose of 291 consistent with diabetes. Blood smears were positive for *P. falciparum* malaria at 9.6% and reticulocyte count was reported at 3.2%. New York City's Department of Health and Mental Hygiene (DOHMH), was contacted and Ebola was not considered to be in Togo, most likely diagnosis was malaria from chloroquine resistant region. Patient was started on quinine 648 mg and doxycycline 100 mg, intravenous (IV) fluids, Lantus 21 U, Lispro 7 U, and was monitored in telemetry unit of medicine (Figures 1–3).

Attention was drawn to the patient at 4:45 am on her 3rd hospital course day after becoming suddenly dyspneic. Patient denied any chest pain but upon pulmonary examination bilateral coarse crackles were heard up to mid lung level. Patient received 60 mg intravenous push (IVP) Lasix and sublingual nitroglycerin. She continued to be dyspneic and was given additional 40 mg IV Lasix and 4 mg Morphine IV were given. Bi-continuous positive airway pressure was started but patient did not tolerate well and decision was made to intubate the patient for acute hypoxemic respiratory failure. Patient was transferred to the medical intensive care unit (ICU) for further care.

Chest X-ray in the medical ICU revealed bilateral alveolar infiltrates; patient was started on Cefepime 2 g IV. Presumption was made that patient had Acute Respiratory Distress Syndrome secondary to sepsis from an unknown source of infection, but possibly from Falciparum Malaria. Abdominal ultrasound showed tiny echogenic foci within the gallbladder, prominent liver measuring 18.7 cm, and a dilated common bile duct measuring 8.2 mm. Choledocholithiasis was questioned although not directly visualized. Decision was made to monitor liver enzymes and if worsening of abdominal status cholecystostomy tube could be placed.

Patient remained in the medical ICU where she was daily monitored. Vital signs monitoring showed daily fever spikes of 101 to 103 °F 2 to 3 times per day. Liver enzymes were down trending after week 1, repeat right upper quadrant ultrasound was negative most probably from passage of a gallstone. On day 9 of hospital course patient was extubated and transferred to medical floors for continuation of care.

## DISCUSSION

Patients of Western African descent presenting with symptoms of fevers and fatigue must be approached with precaution in present day circumstances. The Ebola virus disease outbreak has currently heightened healthcare professional's fears of contracting the virus by exposure to their patients. Furthermore, the impact of the Ebola virus disease in West Africa has left the local population vulnerable to other deadly diseases such as malaria. Control efforts for disease transmission and treatment of malaria have come to a halt. Anti-malaria medication, preventive insecticide bed nets are lying in warehouses far from the people which could benefit from them. International agencies such as the World Health Organization (WHO), US Agency for International Development supported and funded programs malaria control initiatives have virtually been shut down.^5^ The similarities of the symptoms and signs of presentation of both diseases intimidates people from seeking treatment for fear of being infected with Ebola.

In face of all these difficulties, Ebola control efforts including government education programs partnered with WHO, travel measures has reduced the incidence significantly. Early identification of symptoms, isolation of contacts, and early monitoring and treatment has played a major role in limiting spread of infection of Ebola. This case report illustrates an example of how a patient with recent travel history to West Africa presenting with typical fevers, myalgias, and fatigue could be considered to have either or both diseases.
